# Enhancing Seismic Damage Detection and Assessment in Highway Bridge Systems: A Pattern Recognition Approach with Bayesian Optimization

**DOI:** 10.3390/s24020611

**Published:** 2024-01-18

**Authors:** Xiao Liang

**Affiliations:** Zachry Department of Civil and Environmental Engineering, Texas A&M University, College Station, TX 77843, USA; xliang@tamu.edu

**Keywords:** Bayesian optimization, bridge structures, damage detection and assessment, cumulative intensity, support vector machine

## Abstract

Highway bridges stand as paramount elements within transportation infrastructure systems. The ability to ensure swift recovery after extreme events, such as earthquakes, is a fundamental trait of resilient communities. Consequently, expediting the recovery process necessitates near real-time diagnosis of structural damage to provide dependable information. In this study, a data-driven approach for damage detection and assessment is investigated, focusing on bridge columns—the pivotal supporting elements of bridge systems—based on simulations derived from nonlinear time history analysis. This research introduces a set of cumulative intensity-based damage features, whose efficacy is demonstrated through unsupervised learning techniques. Leveraging the support vector machine, a prominent pattern recognition algorithm in supervised learning, alongside Bayesian optimization with a Gaussian process, seismic damage detection and assessment are explored. Encouragingly, the methodology yields high estimation accuracies for both binary outcomes (indicating the presence of damage or the occurrence of collapse) and multi-class classifications (indicating the severity of damage). This breakthrough opens avenues for the practical implementation of on-board sensor computing, enabling near real-time damage detection and assessment in bridge structures.

## 1. Introduction

According to ASCE infrastructure report card [1], the majority of infrastructures in the US are currently rated as mediocre to poor, with many nearing or surpassing their initial design service life and showing signs of deterioration. Among these infrastructures, reinforced concrete (RC) highway bridge systems play a crucial role in transporting goods and people across natural terrains. Ensuring proper recovery after extreme events like earthquakes is vital for building resilient communities. The effective management of post-disaster consequences requires reliable information about the impact of seismic events, thereby necessitating the allocation of existing resources and skills [2]. A resilient system is characterized by its ability to achieve “reduced time to recovery” [3]. Consequently, rapid condition monitoring of highway bridges is imperative [4], with an emphasis on near real-time assessment of structural integrity to determine their safety for reoperation. Traditionally, visual inspections have been the primary method for post-disaster condition assessment. However, deploying dedicated teams for manual inspection poses challenges in terms of both time efficiency and financial resources. Significant efforts have been made to automate the visual inspection process (e.g., [5,6,7,8]). Despite these advancements, automated inspection methods can only detect visible larger defects, leaving the possibility of serious invisible defects going unnoticed [9].

Vibration records serve as another valuable source of information in Structural Health Monitoring (SHM), operating under the assumption that dynamic properties and responses undergo changes in the presence of damage. This approach has been studied extensively over the years (e.g., [10,11]). The model-based approach treats SHM as an inverse problem, utilizing the finite element method and model updating for analysis (e.g., [12,13,14,15,16]), which, however, poses challenges for near real-time implementation. More recent developments in SHM involve data-driven methods. Worden et al. [17] applied outlier analysis to detect damage in a three-degree-of-freedom spring system, utilizing Mahalanobis squared distance as the discordancy measure. Santos et al. [18] explored kernel-based algorithms for damage detection under varying operational and environmental conditions. A hybrid approach based on the expectation-maximization algorithm and Gaussian mixture models was proposed by the same group [19] to identify the normal state of a bridge. Another combined approach, utilizing the concept of symbolic data analysis [20], was applied for structural modification assessment using vibration data from a continuously monitored bridge structure. Abdeljaber et al. [21] proposed the use of one-dimensional convolutional neural networks for damage detection, validated on a grandstand simulator. Other researchers have successfully implemented and applied auto-associative neural networks (e.g., [22,23,24]), auto-regressive models (e.g., [25]), and cluster analysis (e.g., [26,27]) for damage detection in recent years. Modal identification and model updating have also been studied using a Bayesian model (e.g., [28,29,30]).

The aforementioned works exemplify damage detection under operational events. In earthquake engineering, there is significant interest in the rapid condition monitoring of structural health for post-earthquake safety assessment. González and Zapico [31] introduced a seismic damage detection method based on artificial neural networks (ANNs) for buildings with steel moment-frame structures. De Lautour and Omenzetter [32] presented an approach using ANNs to identify seismic-induced damage in two-dimensional (2D) reinforced concrete frames. Elwood et al. [33] proposed an approach based on fuzzy pattern recognition for seismic damage detection in concrete building structures. Zhang et al. [34] employed regression trees and random forests to map building response and damage patterns to residual collapse capacity. Recent methods include using convolutional neural networks [35,36,37] and hybrid deep learning models [38]. Notably, most research efforts in seismic damage detection have focused on 2D building structures subjected to unidirectional ground motion excitation.

This paper introduces a data-driven methodology for damage detection and assessment, utilizing acceleration data obtained from over 60,000 nonlinear time history analysis (NTHA) simulations conducted on two representative RC highway bridge systems subjected to bidirectional GM inputs. This study puts forth a set of low-dimensional cumulative intensity-based damage features, including fractional ones, specifically tailored for bridge columns, which are pivotal components of RC highway bridge systems. The effectiveness of these features is evidenced through their estimated joint probability density function (PDF). A comparative analysis is carried out on selected representative bridge systems under different conditions: normal circumstances and earthquake scenarios with probabilities of exceedance (POEs) of 50%, 10%, and 2% in 50 years, respectively.

This study leverages the support vector machine (SVM), a widely recognized pattern recognition algorithm, to scrutinize structural damage features. The SVM plays a pivotal role in identifying collapse occurrences, detecting damage presence, and assessing severity. Addressing the challenge of overfitting, hyperparameter tuning is conducted through Bayesian optimization [39], wherein the generalization performance of the learning algorithm is modeled as a Gaussian process (GP) sample. To the author’s knowledge, this paper marks the pioneering application of SVM with Bayesian optimization in SHM for civil infrastructures. The outcomes demonstrate highly promising accuracies and robustness (to a significant amount of noise) in both binary (indicating the presence of damage or collapse) and multi-class (indicating the severity of damage) classifications. This research opens up possibilities for leveraging onboard sensor computing, enabling near real-time damage detection and assessment.

## 2. Damage Feature Extraction

**Figure 1:**
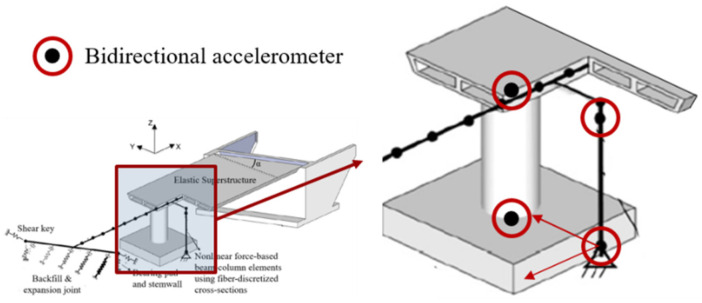
Virtual accelerometers placement on the bridge columns.

An ideal damage feature is a low-dimensional quantity extracted from the system response data, demonstrating a robust correlation with the structural damage state. In this study, the SHM process is simulated by emulating the placement of four accelerometers (virtual sensors) on the bridge column, as illustrated in Figure 1. Among these, two sensors capture the bidirectional ground motion (GM) excitation, while the other two record the acceleration time histories of the column’s top in both longitudinal and transverse directions.

**Figure 2:**
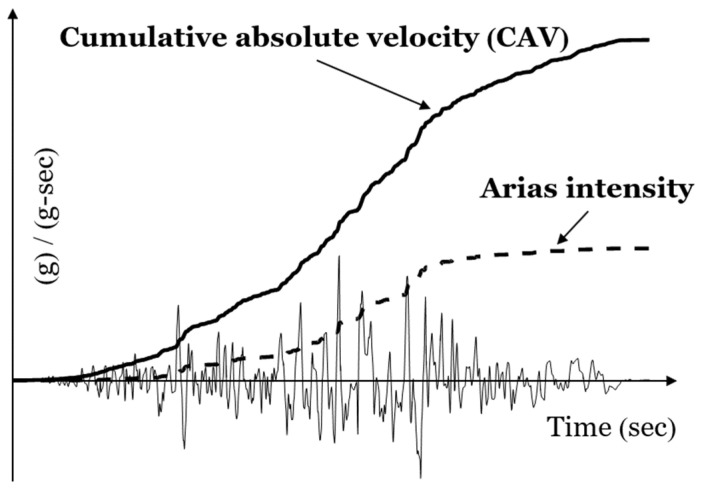
Cumulative absolute velocity (η=1) and Arias intensity (η=2).

It is to be noted that these features serve as the parameters that machine learning algorithms will analyze to identify and quantify damage. Consequently, these damage features are ideally expected to exhibit a monotonic change as the damage levels increase. In this paper, a set of cumulative intensity-based damage features is proposed as follows:

(1) $$I_{\eta }^{g}=\int_{0}^{T_{d}}{\left|a_{g}\left(t\right)\;\right|}^{\eta }\;dt$$

where $a_{g}\left(t\right)$ represents the acceleration time history of the GM input and $T_{d}$ denotes the duration of the earthquake. Consequently, this series of damage features incorporates both amplitude and temporal contributions. It is noted that η=1 and η=2, respectively, lead to cumulative absolute velocity (previously proposed as a damage feature in [40]) and Arias intensity (multiplied by a constant π/2g), as illustrated in Figure 2. The damage feature suggested in Equation (1) holds broader applicability, as η can be any positive real number, thereby eliminating the constraint of being a positive integer. This flexibility allows for the incorporation of potential damage features based on fractional cumulative intensity. To provide a comprehensive assessment of the bridge column’s damage conditions, an additional related feature is introduced as follows:

(2) $$R_{\eta }=\frac{I_{\eta }^{ct}}{I_{\eta }^{g}}=\frac{\int_{0}^{T_{d}}{\left|a_{ct}\left(t\right)\;\right|}^{\eta }\;dt}{\int_{0}^{T_{d}}{\left|a_{g}\left(t\right)\;\right|}^{\eta }\;dt}$$

where $a_{ct}\left(t\right)$ represents the acceleration time history sensed at the bridge column top. When analyzing the bridge column as an input–output system from an energy perspective, the ratio shows a decreasing trend with higher energy dissipation, signifying an escalation in the acquired damages on the bridge column. Nevertheless, normalizing absolute intensity measures through the calculation of the corresponding ratio in Equation (2) leads to the loss of information regarding the magnitude of the input energy. Taking into account that $I_{\eta }^{g}$ corresponds to the input energy of excitation [41], it is prudent to incorporate both absolute and relative intensity measures as inputs. As a result, considering the bidirectional GM input in *x* and *y* directions, for each selected η, a total of four damage features are taken into account in this study, i.e., $I_{\eta -x}^{g},\;I_{\eta -y}^{g},\;R_{\eta -x}$ and $R_{\eta -y}$.

## 3. Pattern Recognition

A pattern recognition algorithm is one that assigns a class label to a sample of measured data, typically by training a diagnostic. Supervised learning algorithms, a category within pattern recognition, educate the diagnostic by presenting it with the true label for each dataset. Consequently, these learning algorithms are crucial for evaluating factors such as the severity of damage, where datasets representing various damage states are employed for training and classification purposes. In this investigation, the use of support vector machine (SVM), a prominent representative of supervised learning algorithms, is explored.

### 3.1. Support Vector Machine

The aim of using the SVM is to construct a hyperplane as defined in the following equation to separate two different classes of data samples ($y_{i}\in \left\{-1,\;1\right\}$) and to maximize the margin from the hyperplane to the closest data points in either class:

(3) $$f\left(x\right)=h{\left(x\right)}^{T}\beta +\beta_{0}=0$$

where x denotes the selected damage features, i.e., $I_{\eta -x}^{g},\;I_{\eta -y}^{g},\;R_{\eta -x}$ and $R_{\eta -y}$ for each η. This hyperplane is in terms of the extended features $h\left(x\right)$. Accordingly, the optimization problem for SVM can be expressed as follows [42]:

(4) $$\begin{array}{c} \underset{\beta ,\;\beta_{0}}{\min}\;\frac{1}{2}{\left\|\beta \right\|}^{2}+C\sum_{i=1}^{N}\xi_{i}\\ s.t.\;\xi_{i}\geq 0,\;y_{i}\left(h{\left(x_{i}\right)}^{T}\beta +\beta_{0}\right)\geq 1-\xi_{i}\;\forall i \end{array}$$

where N is the total number of sampled points, C is the cost parameter to control the tradeoff of bias and variance, and ξ is the slack variable to allow for some data points to be on the wrong side of the margin. The solution to Equation (4) changes Equation (3) into the following:

(5) $$\begin{array}{cc} f\left(x\right) & =h{\left(x\right)}^{T}\beta +\beta_{0}\\ & =\sum_{i=1}^{N}\alpha_{i}y_{i}K\left(x,x_{i}\right)+\beta_{0} \end{array}$$

In this paper, the radial basis kernel function as in Equation (8) is used.

(6) $$K\left(x,x\prime \right)=\exp\left(-\gamma {\left\|x-x\prime \right\|}^{2}\right)$$

### 3.2. Bayesian Optimization

To avoid overfitting, the common practice is to minimize the K-fold cross-validated (CV) [42] loss ($CVL_{K}$) of the SVM model with respect to its hyperparameters, the cost parameter C and the kernel scale γ in this study. First, one splits the training set into *K* non-overlapping subsets. For k=1,2,…,K, the test set is represented by the *k*-th subset, while the training set is represented by the remaining *K*–1 subsets. For the *k*-th iteration, the loss $E_{K}\left(\lambda \right)$ is evaluated, with $\lambda =\left(C,\;\gamma \right)$, while the K-fold CV loss is computed as follows:

(7) $$CVL_{K}\left(\lambda \right)=\frac{1}{K}\sum_{k=1}^{K}E_{k}\left(\lambda \right)$$

The objective function at hand is evidently non-convex, lacking a closed-form expression, and thus its derivatives are inaccessible. One can only acquire observations of this function at sampled values, and such evaluations come at a considerable cost. Consequently, direct application of common optimization algorithms, like the Monte Carlo method or Genetic Algorithm, appears impractical. Bayesian optimization emerges as a potent strategy for extremum discovery in cases where the objective function, such as the one presented in Equation (10), is difficult to assess. What sets Bayesian optimization apart is its approach: it constructs a probabilistic model for the objective function and utilizes this model to determine the next point for evaluation. The aim is to leverage all available information from previous evaluations, thus avoiding an exclusive reliance on local gradient and Hessian approximations [39]. Despite the additional computational effort required to determine the next point for evaluation, Bayesian optimization generally proves to be effective in identifying the minimum of challenging non-convex functions with relatively few evaluations [43].

The fundamental assumption adopted in Bayesian optimization is that the function $CVL_{K}\left(\lambda \right)$ is drawn from a GP prior, i.e., $CVL_{K}\left(\lambda \right)\sim N\left(0,K\right)$ (without loss of generality, the prior mean is given as 0), whose kernel matrix is given by

(8) $$K=\left[\begin{array}{ccc} k\left(\lambda_{1},\lambda_{1}\right) & \ldots & k\left(\lambda_{1},\lambda_{t}\right)\\ \vdots & \ddots & \vdots \\ k\left(\lambda_{t},\lambda_{1}\right) & \ldots & k\left(\lambda_{t},\lambda_{t}\right) \end{array}\right]$$

where $k\left(\lambda ,\lambda^{\prime }\right)$ is the covariance function. From previous iterations, the following observation is acquired: $D_{1:t}=\left\{\lambda_{1:t},CVL_{1:t}^{K}\right\}$, where $CVL_{1:t}^{K}=CVL_{K}\left(\lambda_{1:t}\right)$. $\lambda_{t+1}$ is obtained as the next point to evaluate and denote the value of the function at $\lambda_{t+1}$ as $CVL_{t+1}^{K}=CVL_{K}\left(\lambda_{t+1}\right)$. Under the GP prior, $CVL_{1:t}^{K}$ and $CVL_{t+1}^{K}$ are jointly Gaussian and one can obtain the following expression for the predictive distribution [39,44]:

(9) $$CVL_{t+1}^{K}|D_{1:t}\sim N\left(\mu \left(\lambda_{t+1}\right),\sigma^{2}\left(\lambda_{t+1}\right)\right)$$

where

(10) $$\mu \left(\lambda_{t+1}\right)=k^{T}K^{-1}CVL_{1:t}^{K}$$

(11) $$\sigma^{2}\left(\lambda_{t+1}\right)=k\left(\lambda_{t+1},\lambda_{t+1}\right)-k^{T}K^{-1}k$$

(12) $$k={\left[\begin{array}{cccc} k\left(\lambda_{t+1},\lambda_{1}\right) & k\left(\lambda_{t+1},\lambda_{2}\right) & \cdots & k\left(\lambda_{t+1},\lambda_{t}\right) \end{array}\right]}^{T}$$

Therefore, the predictive posterior distribution $CVL_{t+1}^{K}|D_{1:t}$ is sufficiently characterized by its predictive mean function $\mu \left(\lambda_{t+1}\right)$ and predictive variance function $\sigma^{2}\left(\lambda_{t+1}\right)$, which solely depend on the selection of the covariance function $k\left(\lambda ,\lambda^{\prime }\right)$. In this study, the automatic relevance determination (ARD) Matérn 5/2 kernel recommended in [43] is used as follows to permit greater flexibility in modeling function:

(13) $$k_{M52}\left(\lambda ,\lambda^{\prime }\right)=\theta_{0}\left[1+\sqrt{5r^{2}\left(\lambda ,\lambda^{\prime }\right)}+\frac{5}{3}r^{2}\left(\lambda ,\lambda^{\prime }\right)\right]\exp\left(-\sqrt{5r^{2}\left(\lambda ,\lambda^{\prime }\right)}\right)$$

where

(14) $$r^{2}\left(\lambda ,\lambda^{\prime }\right)=\sum_{d=1}^{D}{\left(\lambda_{d}-\lambda_{d}^{\prime }\right)}^{2}/\theta_{d}^{2}$$

where $\theta_{0}$ and $\theta_{d},\;d=1,\ldots ,D$, are the hyperparameters of the ARD Matérn 5/2 kernel that are learned by “seeding” with a few random samples and maximizing the log-likelihood of the evidence given $\theta =\left(\theta_{0},\theta_{1},\ldots ,\theta_{D}\right)$ [32,36]. In this case, D=2 corresponds to the dimensionality of $\lambda =\left(C,\;\gamma \right)$.

To sample efficiently, Bayesian optimization uses an acquisition function to determine the next location $\lambda_{t+1}$ for evaluation. The acquisition function used in this study is the Expected Improvement (EI), which is to maximize the EI over the best current value $\lambda_{best}=\arg\min_{\lambda_{i}\in \lambda_{1:t}}CVL_{K}\left(\lambda_{i}\right)$. This has a closed-form solution under the GP [44] assumption as follows:

(15) $$a_{\mathrm{EI}}\left(\lambda_{t+1}\right)=\sigma \left(\lambda_{t+1}\right)\left[Z\;\Phi \left(Z\right)+\phi \left(Z\right)\right]$$

where

(16) $$Z=\frac{CVL_{K}\left(\lambda_{best}\right)-\mu \left(\lambda_{t+1}\right)}{\sigma \left(\lambda_{t+1}\right)}$$

and $\Phi \left(\cdot \right)$ and $\phi \left(\cdot \right)$, respectively, denote cumulative distribution function and PDF of the standard normal. Unlike the original unknown objective function in Equation (7), $a_{\mathrm{EI}}\left(\cdot \right)$ can be cheaply sampled to be maximized. Note that GPs scale cubically with the number of observation; in summary, the goal of Bayesian optimization is to efficiently discover the global optimum with a limited number of evaluations by intelligently allocating additional computing power to identify the next point for assessment. The algorithm of SVM with Bayesian optimization is summarized in Algorithm 1.
**Algorithm 1** SVM with Bayesian optimization**for** t=1,2,… **do** 1. Calculate predictive mean function $\mu \left(\lambda_{t+1}\right)$ and predictive variance function $\sigma^{2}\left(\lambda_{t+1}\right)$ using the selected kernel function $k_{M52}\left(\lambda ,\lambda^{\prime }\right)$2. Find $\lambda_{t+1}=\left(C_{t+1},\;\gamma_{t+1}\right)$ by optimizing the acquisition function over the GP:$\lambda_{t+1}=\arg\max_{\lambda }a_{\mathrm{EI}}\left(\lambda |D_{1:t}\right)$3. With SVM parameterized by $C_{t+1}$ and $\gamma_{t+1}$, evaluate the objective function: $CVL_{K}\left(\lambda_{i+1}\right)$4. Augment the data $D_{1:t+1}=\left\{D_{1:t},\left(\lambda_{t+1},CVL_{t+1}^{K}\right)\right\}$ and update the GP**end for**

The proposed pattern recognition algorithm is implemented through the following steps:

1. Generate damage features from the training data, as detailed in Section 2;
2. Train and fine-tune support vector machines (SVMs) according to the procedures outlined in Section 3. This involves using the generated damage features from Step 1 along with corresponding labels (e.g., damaged or not);
3. Employ the trained SVMs from Step 2 for future predictions when a new set of acceleration records is acquired.

## 4. Case Study

In this section, the proposed framework is investigated on RC highway bridge systems.

### 4.1. Computational Bridge Model and Ground Motion Selection

Two representative RC highway bridge systems (designed after 2000), Jack Tone Road Overcrossing (denoted as Bridge A) and La Veta Avenue Overcrossings (denoted as Bridge B), are selected for this study. Comprehensive analytical modeling and simulations of these bridges can be found in [45]. The software platform OpenSees (version 3.5.0) [46] is employed for both the modeling and simulations. The computational models explicitly encompass the superstructure, column-bents, and seat-type abutments. Given that modeling assumptions can significantly influence the dynamic response characteristics of short bridges [47,48], verified and/or validated modeling techniques are adopted whenever feasible.

**Figure 3:**
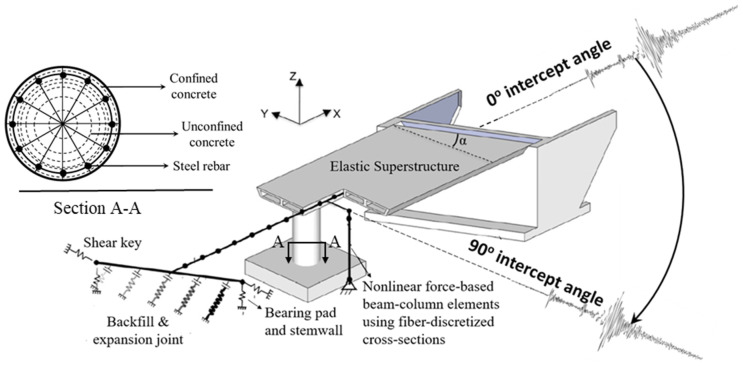
Modeling of the bridge under bidirectional GM input considering different intercept angles.

The bridge superstructure (depicted in Figure 3), comprising the bridge deck and cap beam, is modeled using elastic beam–column elements with uncracked section properties. Exceptionally high torsional and out-of-plane stiffness values are assigned to the cap beam due to its integral construction with the deck. To accurately capture dynamic responses, the mass of the superstructure, including rotational mass, is distributed to the superstructure elements. The bridge column is represented by nonlinear force-based beam–column elements (as illustrated in Figure 3), incorporating fiber-discretized cross-sections. This approach employs three concurrent constitutive models: (1) confined concrete for the core, (2) unconfined concrete for the cover, and (3) steel for the reinforcing bars. For both cover and core concretes, following the methodology in [49], the Concrete01 constitutive model is applied. This model represents a uniaxial Kent–Scott–Park concrete material object with degraded linear unloading/reloading stiffness and no tensile strength. The steel reinforcing bars are modeled using the Steel02 material, which represents a uniaxial Giuffre–Menegotto–Pinto steel material object with isotropic strain hardening [50].

**Figure 4:**
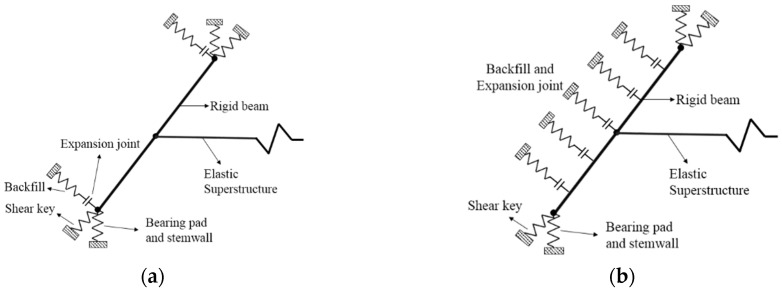
Abutment modeling with springs and gap elements. (**a**) Type I. (**b**) Type II.

Two modeling approaches, designated as Type I and Type II, are under consideration for the abutment (refer to Figure 4). Both approaches explicitly address longitudinal, transverse, and vertical responses. In Type I (as illustrated in Figure 4a), the model employs two nonlinear springs, each located at the ends, connected in series to gap elements. These springs, modeled with an elastic-perfectly plastic (EPP) backbone, represent the passive backfill response and the expansion joint, respectively [51]. The transverse direction incorporates an EPP backbone relationship to model the backfill–wingwall–pile system, with the Type I model ignoring the resistance of the shear keys for simplicity. The vertical response of the bearing pads and stemwall is captured using two parallel springs. The first spring represents the flexible part of the elastomeric bearing pad in the vertical direction, and the second represents the vertical stiffness of the stemwall. In Type II (depicted in Figure 4b), the longitudinal response is modeled using five abutment nonlinear hyperbolic springs connected in series to gap elements. Additionally, the resistance provided by the shear key is modeled in the transverse direction using a nonlinear spring with a tri-linear backbone relationship. It is noteworthy that the modeling technique employed in this study aligns with the approach utilized by Cruz and Saiidi [52], validated through a large-scale four-span bridge test at the University of Nevada, Reno, demonstrating a comparable correlation between seismic demands derived from analytical models and experimental data.

Utilizing a magnitude 7 earthquake scenario outlined in [53], 99 pairs of seed bidirectional horizontal ground motion (GM) records are selected from the PEER Next Generation Attenuation (NGA) Project GM database [54]. Subsequently, these 99 pairs of GM records are scaled based on the lognormal distribution of peak ground velocity (PGV) as detailed in [55]. For this investigation, 25 PGV values (representing 25 intensity levels) to encompass this distribution are chosen. Additionally, various intercept angles, ranging from 0 to 150 in increments of 30 (refer to Figure 3), are explored. Consequently, considering both Bridges A and B, abutment modeling Types I and II, and the six intercept angles mentioned above for all 99 unscaled GMs with 25 PGV values, a total of 59,400 NTHA simulations are conducted. In this extensive set, simulations for the first five intercept angles are designated for training, while those for the last intercept angle constitute the test set. Both sets comprise representative samples, including damaged and undamaged instances, addressing the classification problem related to the existence of damage.

### 4.2. Damage Feature

The efficacy of $R_{\eta }$ proposed in Equation (2) as a damage feature is demonstrated through unsupervised learning, where a statistical model (such as a joint Probability Density Function) of damage features during the undamaged state is established [56,57]. Monitoring data are then compared against this model. In this study, the multivariate probabilistic model of Distributions with Independent Components [58,59] is employed, with univariate distributions modeled through kernel density estimation (KDE) [60]. The dataset is derived from 99 ground motions with small scaling factors, ranging from 0.01 to 0.1 in increments of 0.01. This dataset comprises a total of 990 NTHA simulations, representing undamaged conditions for each investigated bridge system configuration (e.g., Bridge A with Type I abutment modeling). The detailed procedure is outlined in the Appendix A.

**Figure 5:**
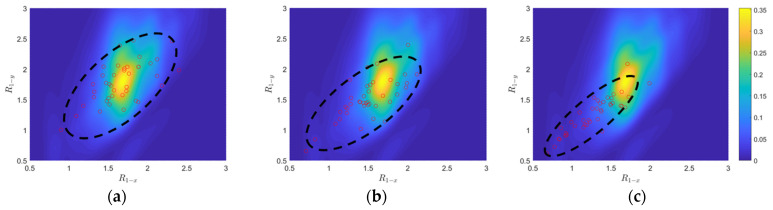
Comparisons between joint PDF of R_1−x_ versus R_1−y_ for the undamaged condition and R_1−x_ versus R_1−y_ of three damage levels: 50%, 10%, and 2% POE in 50 years. (**a**) 50% POE. (**b**) 10% POE. (**c**) 2% POE.

**Figure 6:**
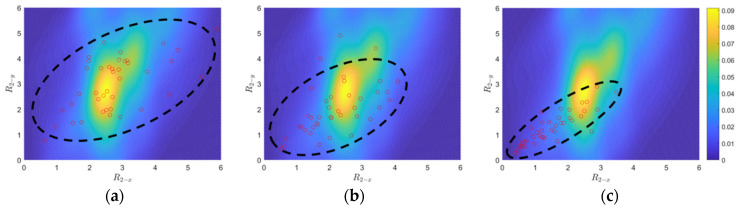
Comparisons between joint PDF of R_2−x_ versus R_2−y_ for the undamaged condition and R_2−x_ versus R_2−y_ of three damage levels: 50%, 10%, and 2% POE in 50 years. (**a**) 50% POE. (**b**) 10% POE. (**c**) 2% POE.

Three sets of 40 GMs, which correspond to the earthquake scenarios with 50%, 10%, and 2% POE in 50 years, are selected to represent three damage levels for the bridges. As a demonstration, Figure 5 and Figure 6, respectively, show the comparisons between the joint PDF (the heat maps) and the three groups for $R_{1}$ and $R_{2}$ (red dots), respectively. It is noted that the joint PDF of $R_{2}$ is much flatter than that of $R_{1}$ (e.g., peaks from 1.6 to 1.8 for $R_{1-x}$ compared to those from 2.3 to 2.8 for $R_{2-x}$), which explains the order of magnitude difference between their color bars. As the damage level increases (i.e., from 50% POE in 50 years to 10% POE in 50 years, and then to 2% POE in 50 years), clear monotonic trends are discernible for both damage features (as groups), showcasing a gradual shift of ellipses (encompassing most of the red dots), toward the left lower corner. Consequently, $R_{1}$ and $R_{2}$ are effective damage indicators for bridge column of the investigated RC highway bridge systems and can be used as damage features in the pattern recognition algorithm introduced next.

### 4.3. Simulated Measurement Noise

**Figure 7:**
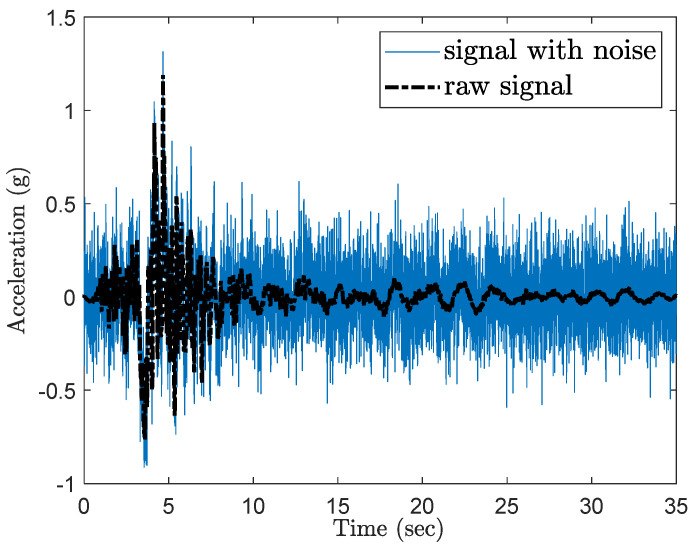
Time history reading of the column top for one GM scaled to the highest intensity level.

For earthquake event applications, it is crucial that the damage detection and assessment algorithm remains robust in the presence of measurement noise in sensor recordings. To simulate such noise, the following procedures are proposed:

1. Random Gaussian noise, with a noise-to-signal ratio of 30% (calculated as the ratio of standard deviations within the duration of each ground motion), is added to the acceleration time history of the input GM excitation at the column bottom (see Figure 1);
2. The acceleration time history at the column top is obtained by summing up the acceleration at the ground level (with noise) from Step 1 and the relative acceleration from NTHA simulations. Note that the relative acceleration is recorded in OpenSees [48] for the column top;
3. Again, random Gaussian noise with a noise-to-signal ratio of 30% is added to the obtained acceleration time history at the column top in Step 2. Figure 7 illustrates the comparison of the acceleration signal at the column top with and without noise for one GM scaled to the highest intensity level.

**Figure 8:**
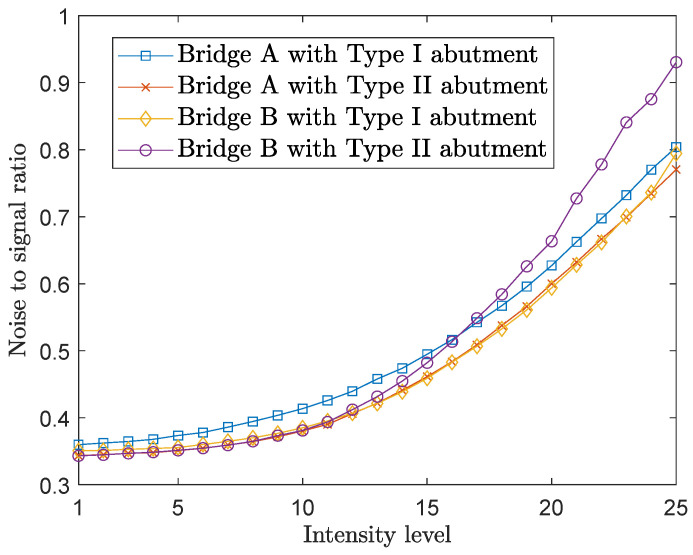
The average noise-to-signal ratio of the four bridge configurations under all intensity levels for the 30-degree intercept angle.

Following these procedures, for each GM, the noise-to-signal ratio consistently increases with the increase in intensity level. Figure 8 depicts such trends, presenting the average noise-to-signal ratio for the selected 99 ground motions across all four bridge configurations for the 30-degree intercept angle.

### 4.4. Classification Results

**Figure 9:**
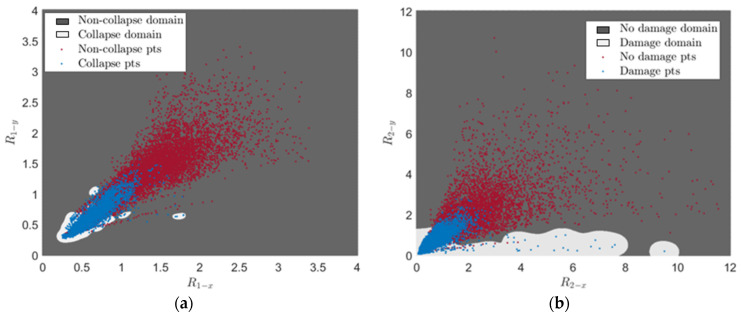
SVM training results for the occurrence of collapse using R_1−x_ and R_1−y_ and existence of damage using R_2−x_ and R_2−y_ for Bridge A with Type I abutment modeling. (**a**) R_1−x_ and R_1−y_. (**b**) R_2−x_ and R_2−y_.

**Figure 10:**
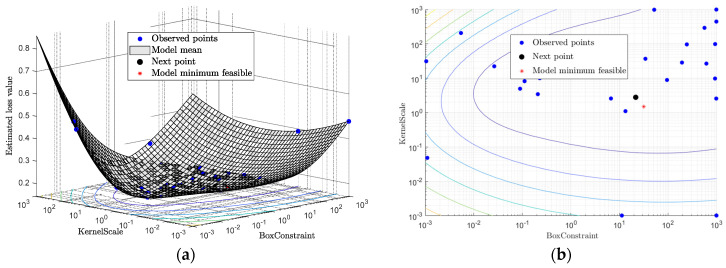
An illustration of cross-validated loss minimization using Bayesian optimization and its bird view. (**a**) Bayesian optimization. (**b**) Bird view.

For visualization purposes, Figure 9 shows the training results using $R_{1}$ and $R_{2}$, (refer to Equation (2)), respectively, as damage features for predicting the occurrence of collapse (i.e., when the peak column drift ratio exceeds 8% [61]) and the existence of damage (i.e., when the peak column drift ratio exceeds 2% [62]) for Bridge A with Type I abutment modeling. The decision boundary is determined by the labeled training data and an SVM tuned using Bayesian optimization. It is important to note that the decision boundary is nonlinear, and the damage regions exhibit discontinuities due to the utilization of a nonlinear kernel, as defined in Equation (6). Figure 10 illustrates the minimization of the 10-fold cross-validated loss (adopted as the objective function in this paper) using Bayesian optimization. Both classification tasks—detecting the existence of damage and predicting the occurrence of collapse—are performed for all investigated bridge configurations using the damage features outlined in Table 1. In this study, the damage feature vector is of around ten dimensions [4]. As mentioned earlier, for each η, a total of four damage features, i.e., $I_{\eta -x}^{g},\;I_{\eta -y}^{g},\;R_{\eta -x}$ and $R_{\eta -y}$, are considered. Consequently, only two or three values of η are taken into account. The values of η in Table 2 are selected based on the minimum 10-fold cross-validated loss achieved by performing SVM with Bayesian optimization under different two and three η combinations. It is found that the loss is smaller for all investigated classification cases when three values of are considered. Therefore, 12 damage features serve as the input for the SVM classifiers.

**Figure 11:**
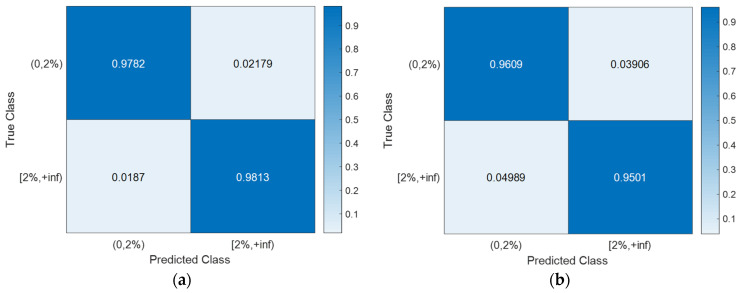
Existence of damage: confusion matrices for Bridge B with Type I abutment modeling. (**a**) Training set. (**b**) Testing set.

**Figure 12:**
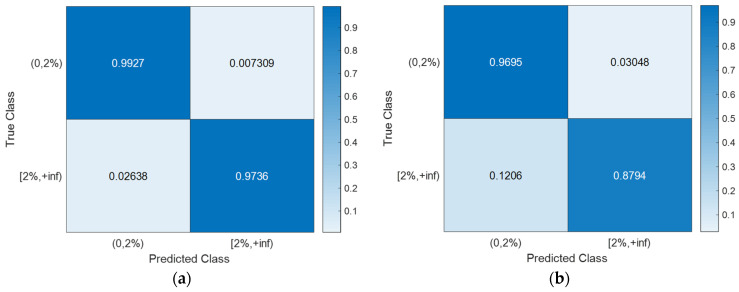
Occurrence of collapse: confusion matrices for Bridge B with Type II abutment modeling. (**a**) Training set. (**b**) Testing set.

**Figure 13:**
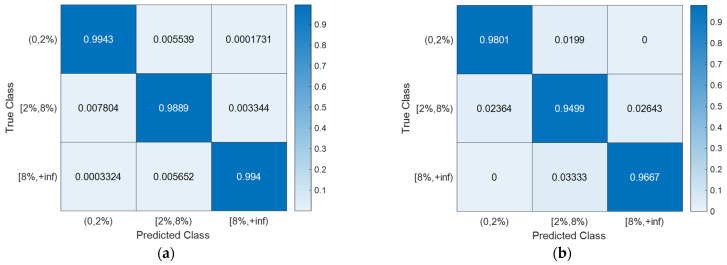
Severity of damage: confusion matrices for Bridge A with Type I abutment modeling. (**a**) Training set. (**b**) Testing set.

**Figure 14:**
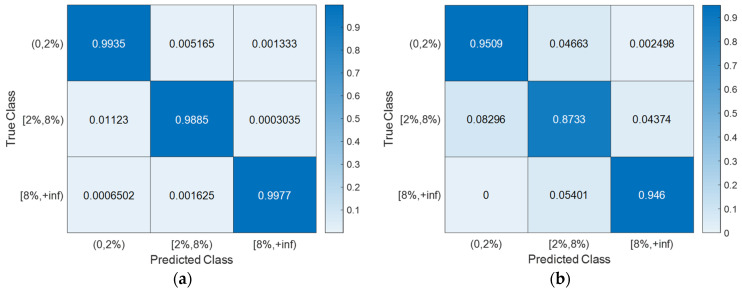
Severity of damage: confusion matrices for Bridge A with Type II abutment modeling. (**a**) Training set. (**b**) Testing set.

**Figure 15:**
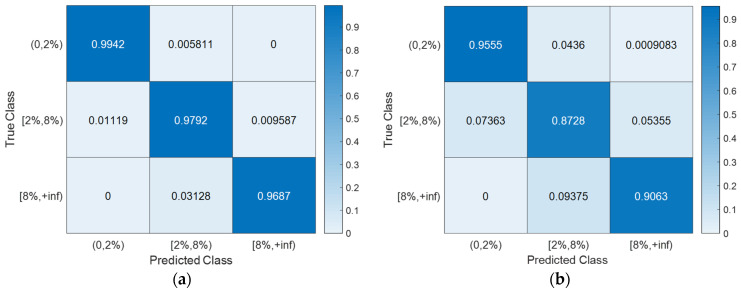
Severity of damage: confusion matrices for Bridge B with Type I abutment modeling. (**a**) Training set. (**b**) Testing set.

**Figure 16:**
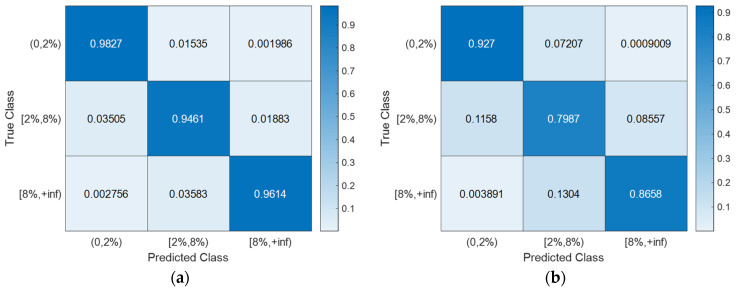
Severity of damage: confusion matrices for Bridge B with Type II abutment modeling. (**a**) Training set. (**b**) Testing set.

With the hyperparameter values of the SVM models (Table 3) determined using Bayesian optimization (searching over a cube with $C,\;\gamma \in \left[0.001,1000\right]$, as shown in Figure 8), the training, CV, and testing accuracies for all scenarios are documented in Table 4. Notably, the CV accuracy (i.e., 1-$CVL_{K}$; note that $CVL_{K}$ is the cost function as in Equation (9) for minimization) closely approximates the testing accuracy. While a subtle decrease in testing accuracy is observed with more intricate structures—from single-column Bridge A to two-column Bridge B and from Type I abutment modeling to Type II with additional springs and gap elements—the testing accuracies remain remarkably high for these two binary classifications. Figure 11 and Figure 12 provide example confusion matrices for both training and testing sets, illustrating accuracies and misclassification errors for each class. The SVM is further extended to handle multi-class classification problems. In this paper, a three-class classification is conducted: no damage, damaged without collapse, and collapse (i.e., peak column drift ratio below 2%, between 2% and 8%, and above 8%). This entails three SVM classifiers, each time comparing one of the three classes to the remaining two. In this case, λ in Algorithm 1 becomes a vector with six elements, representing three cost parameters and three kernel scales-one pair for each SVM classifier. The last two columns of Table 2 contain the hyperparameters values for the three SVM models, i.e., from top to bottom, $\left(0,2\%\right),\;\left[2\%,8\%\right)$, and $\;\left[8\%,+\inf\right)$ versus the remaining two classes. Remarkably, promising accuracies of approximately 90% are achieved (Table 3). Additionally, Figure 13, Figure 14, Figure 15 and Figure 16 present the confusion matrices for training and testing sets in the three cases, thereby providing the predicted accuracies for each class. It is noteworthy that the accuracies for training and testing sets in all cases are comparable, indicating that the Bayesian-optimized SVM classifiers exhibit robustness against overfitting. The advantages of Bayesian optimization are evident in the comparisons of achieved testing accuracies with and without adopting Bayesian optimization for hyperparameter selection (Table 4). It is important to note that the hyperparameters leading to testing accuracies without Bayesian optimization are randomly selected (i.e., those used in the first iteration of the corresponding Bayesian optimization).

## 5. Conclusions

This paper introduces a novel data-driven approach for detecting damage in bridge columns through nonlinear time history simulations conducted on a reinforced concrete highway bridge system. The proposed structural health monitoring method simulates the placement of four accelerometers on the bridge column. Two of these accelerometers measure bidirectional GM excitation, while the other two record the acceleration time histories of the column’s top in both longitudinal and transverse directions. A set of cumulative intensity-based damage features, including fractional ones, is derived from the acceleration time histories. These features have been proven to be effective and reliable indicators of damage through unsupervised learning. The analysis takes into account distributions with independent components, utilizing univariate kernel density distributions. Subsequently, a support vector machine is applied, with its hyperparameters optimized using Bayesian optimization. This approach is used to address various binary and multi-class classification problems related to damage diagnosis, such as predicting the occurrence of collapse, identifying the existence of damage, and assessing its severity. Remarkably high accuracies and robustness are achieved, even when subjected to simulated measurement noise with a high signal-to-noise ratio. This suggests the model’s potential for implementation in sensor networks equipped with onboard computing capabilities, thereby enabling near real-time damage detection and assessment.

## Figures and Tables

**Table 1:** Selected damage features for investigated classification cases.

Bridge	Abutment	η Considered		
		Existence of Damage	Occurrence of Collapse	Severity of Damage
A	I	0.1, 1.0 and 1.5	0.1, 0.5 and 1.5	0.1, 0.5 and 1.5
	II	0.1, 0.5 and 1.0	0.1, 1.5 and 2.0	0.2, 1.0 and 1.5
B	I	0.2, 1.0 and 1.5	0.5, 1.5 and 2.0	0.1, 1.0 and 2.0
	II	0.1, 0.2 and 0.5	0.5, 1.5 and 2.0	0.5, 1.0 and 2.0

**Table 2:** Hyperparameters selected using Bayesian optimization for investigated classification cases.

Bridge	Abutment	Existence of Damage		Occurrence of Collapse		Severity of Damage	
		C	γ	C	γ	C	γ
A	I	113.82	0.97	3.82	0.66	45.11	0.88
						128.88	0.63
						3.91	1.00
	II	234.37	1.64	38.67	2.55	250.66	2.55
						736.63	0.37
						32.86	1.45
B	I	978.48	2.55	19.69	2.28	446.32	1.51
						154.75	3.50
						263.41	4.16
	II	960.34	1.09	9.39	1.34	888.00	7.59
						13.53	1.08
						242.96	6.30

**Table 3:** Training, CV, and testing accuracies achieved for investigated classification cases.

Bridge	Abutment	Existence of Damage			Occurrence of Collapse			Severity of Damage		
		Training	CV	Testing	Training	CV	Testing	Training	CV	Testing
A	I	99%	97%	98%	99%	98%	99%	99%	96%	97%
	II	97%	95%	95%	99%	97%	98%	99%	92%	93%
B	I	98%	95%	96%	99%	96%	96%	98%	92%	92%
	II	96%	93%	93%	99%	94%	95%	97%	87%	88%

**Table 4:** Comparisons of testing accuracy between with and without Bayesian optimization for hyperparameters.

Scenario		Existence of Damage		Occurrence of Collapse		Severity of Damage	
Bayesian Optimization		No	Yes	No	Yes	No	Yes
Bridge A	Type I	73%	98%	76%	99%	61%	97%
	Type II	80%	95%	75%	98%	62%	93%
Bridge B	Type I	64%	96%	76%	96%	63%	92%
	Type II	67%	93%	77%	95%	63%	88%

## Data Availability

Data are contained within the article.

## Appendix A

The procedure of Distributions with Independent Components (DIC) using Kernel Density Estimation (KDE) for the univariate distributions is as follows:

1. Apply Independent Component (IC) analysis to the data X, e.g., $X=\left(R_{1-x},\;R_{1-y}\right)$, in the original space to obtain the data Y in the IC space as follows:
  (A1) $$\begin{array}{l} Y=P\left(X-\mu_{X}\right)\\ X=\mu_{X}+QY \end{array}$$
  where $\mu_{X}$ stands for mean matrix of X and $Q=P^{-1}$.
2. Apply KDE to each IC and the joint PDF in the IC space can be obtained as follows:
  (A2) $$p_{Y}\left(y\right)=p_{Y_{1}}\left(y_{1}\right)\cdot p_{Y_{2}}\left(y_{2}\right)\cdot \ldots \cdot p_{Y_{n}}\left(y_{n}\right)$$
3. The joint PDF in the original space can be obtained as follows with |●| denotes the determinate of the matrix:
  (A3) $$p_{X}\left(x\right)=\frac{1}{\left|Q\right|}p_{Y}\left[P\left(x-\mu_{X}\right)\right]$$
